# Intestinal Mucosal Barrier Is Regulated by Intestinal Tract Neuro-Immune Interplay

**DOI:** 10.3389/fphar.2021.659716

**Published:** 2021-05-31

**Authors:** Xin-yu You, Han-yu Zhang, Xu Han, Fang Wang, Peng-wei Zhuang, Yan-jun Zhang

**Affiliations:** ^1^ Tianjin Key Laboratory of Chinese medicine Pharmacology, Tianjin University of Traditional Chinese Medicine, Tianjin, China; ^2^ State Key Laboratory of Component-based Chinese Medicine, Tianjin University of Traditional Chinese Medicine, Tianjin, China

**Keywords:** norepinephrine, cholinergic anti-inflammatory pathway, enteric nervous system, sensory neurone, immunity, gut microbes

## Abstract

Inflammatory bowel disease, irritable bowel syndrome and severe central nervous system injury can lead to intestinal mucosal barrier damage, which can cause endotoxin/enterobacteria translocation to induce infection and is closely related to the progression of metabolic diseases, cardiovascular and cerebrovascular diseases, tumors and other diseases. Hence, repairing the intestinal barrier represents a potential therapeutic target for many diseases. Enteral afferent nerves, efferent nerves and the intrinsic enteric nervous system (ENS) play key roles in regulating intestinal physiological homeostasis and coping with acute stress. Furthermore, innervation actively regulates immunity and induces inherent and adaptive immune responses through complex processes, such as secreting neurotransmitters or hormones and regulating their corresponding receptors. In addition, intestinal microorganisms and their metabolites play a regulatory role in the intestinal mucosal barrier. This paper primarily discusses the interactions between norepinephrine and β-adrenergic receptors, cholinergic anti-inflammatory pathways, nociceptive receptors, complex ENS networks, gut microbes and various immune cells with their secreted cytokines to summarize the key roles in regulating intestinal inflammation and improving mucosal barrier function.

## Introduction

Function of the digestive tract is controlled by both CNS and ENS, and it is one of the most extensive immune organs in the body, maintaining the balance between immunogenicity and immune tolerance of food, foreign bodies and microorganisms. An increasing number of studies have found that the neuroimmune gut-brain axis has a significant impact on the quality of life and disease outcomes of patients with nonspecific IBD, IBS and functional dyspepsia (FD) and plays an important role in regulating gastrointestinal dysfunction caused by stroke (Huang et al., 2019) and neurodegenerative diseases (Vanuytsel et al., 2014; Han et al., 2017; Campos-Acuna et al., 2019). Peripheral nerves transmit information on the intestinal immune state to the CNS, which regulates the intestinal microenvironment.

Intestinal epithelial cells (IECs) is the first immune barrier against foreign substances or microorganisms. Intestinal stem cells differentiate at the base of the crypts (Bernstein, 2017) to form a variety of IECs, such as Paneth cells, intestinal endocrine cells (Goll and van Beelen Granlund, 2014), M cells (Mabbott et al., 2013; Lai et al., 2019), epithelial cells that absorb columnar, goblet cells that secrete mucus (Bron et al., 2017; Wells et al., 2017; Murai et al., 2018). And the protein complexes composed of tight junctions, adhesive junctions, desmosomes and gap junctions to regulate the function of the intestinal epithelial barrier. Pattern recognition receptors (PRRS) expressed on IEC monitor the dynamic microbial environment and actively participate in the cellular immune response of mucous membrane. Among them, goblet cells and Paneth cells participate in adaptive immunity regulation via transporting and secreting IgA (sIgA) and antimicrobial peptide (AMP). Monocyte phagocytes and antigen-presenting cells (APCs) in the lamina propria of the intestine affect the initiation of cellular and humoral adaptive immune response by communicating with IEC. Persistent intestinal barrier dysfunction, IEC death and inflammation are significant characteristics of IBD. Intestinal cavity antigens pass through the damaged intestinal barrier, resulting in intestinal wall immune activation, but the increase in intestinal mucosal barrier permeability is also the result of inflammatory alterations. Therefore, changes and causal relationship in intestinal mucosal permeability with nerves and immune activation are also the focus of our discussion (Vanuytsel et al., 2014).

A large number of studies have confirmed that nerves regulations or immune responses play a critical role in the repair of intestinal mucosal barrier in IBD. This paper focuses on the crosstalk between Autonomic nervous system (ANS), afferent nerve and ENS and the immune impact on IEC, as well as their protecting role in intestinal mucosal barrier. The gastrointestinal mucosal barrier is dominated by ANS, and the muscle function and blood flow of the mucosa are regulated by sympathetic nerves, which secretion neurotransmitters combine with receptors on intestinal immune cells to affect intestinal mucosal barrier function. While the vagus nerve (VN) indirectly regulates histamine secretion, resulting in increased intestinal mucosal barrier permeability (Li et al., 2020). The VN is responsible for the regulation of the intestinal tract by the parasympathetic nervous systems (PNS), which extends from the brain stem to the intestinal ENS and is the primary nerve connecting the gut-brain axis. Nicotine or electrical stimulation of the VN activates the CAIP and inhibits proinflammatory cytokines without interfering with the expression of anti-inflammatory factors (Báez-Pagán et al., 2015). Among them, intestinal resident muscularis macrophages (MMΦs) can not only prevent neuron apoptosis through upregulating β2-adrenergic receptor (β2-AR) signal, but also exert anti-inflammatory effect upon VN, which a critical part of intestinal mucosal barrier (Meroni et al., 2018; Matheis et al., 2020). Sensory afferent nerves located in the spinal cord and brainstem can release neuropeptides to stimulate mast cell degranulation and regulate the intestinal adaptive immune response. The axonal end of the afferent nerve of the dorsal root ganglion (DRG) extends into the intestinal mucosa, which is the primary monitor of bacterial infectious IBD (Xu et al., 2018). The ENS controls intestinal function independently of the CNS, and enteral glial cells (EGCs) support the ENS network and maintain the integrity of the epithelial barrier. Intestinal motor neurons regulate the production of AMP by secreting neurotransmitters and innate lymphoid cells (ILCs) to protect the intestinal mucosal epithelium (Ibiza et al., 2016; Kermarrec et al., 2018; Talbot et al., 2020). In addition, some intestinal microorganisms and metabolites have been shown to regulate the development and function of exogenous nerves and the ENS, which can bind to toll-like receptors on immune cells to induce an adaptive immune response.

Under background of gut-brain axis interaction, this paper reviews how different branches of the nervous system interacting with the intestinal innate and adaptive immune response can futher modulate the function of intestinal mucosal barrier. Summarizing the symptomatic of bowel infection and intestinal inflammation, would provide reference for the treatment of intestinal mucosal barrier injury, which is the intermediate link of CNS injury and other diseases.

## Intestinal Efferent Nerve-Immunity and Intestinal Mucosal Barrier Function

**FIGURE 1:**
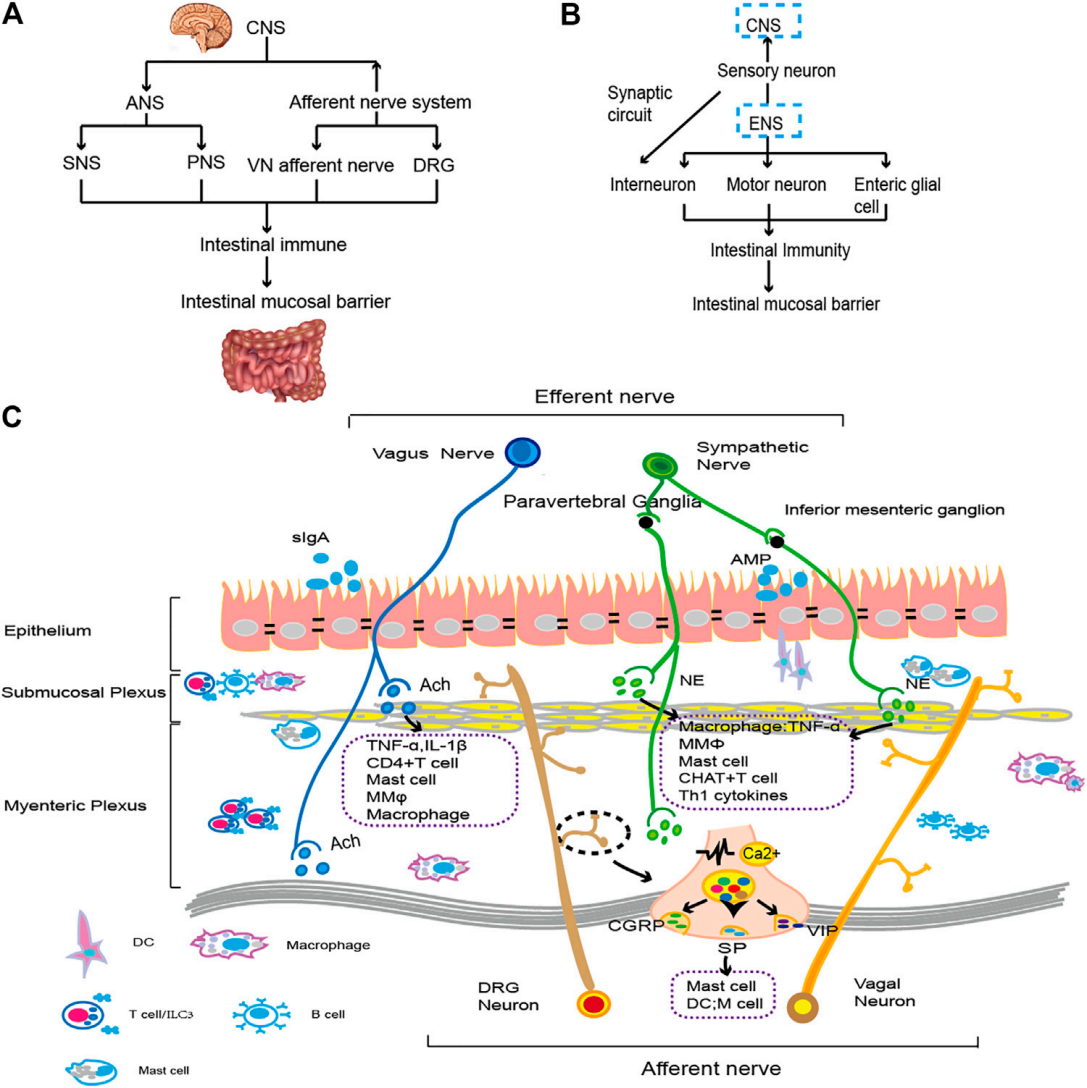
The gut brain axis and neuroimmunity. **(A)**. The CNS connects with the intestine through the ANS and afferent nerves. By regulating immune cells and cytokines, it affects the function of the intestinal epithelial cell barrier. The specific types of immune regulation are shown in **(C)**. **(B)**. The ENS transmits information to the CNS through sensory nerves. Moreover, motor neurons, intermuscular neurons and intestinal glial cells of the ENS interact with intestinal resident or exogenous immune cells and then affect the composition and function of the intestinal mucosal epithelial barrier. The specific immune types are shown in **(C)**. **(C)**. ANS fibers from the CNS extend into intestinal tissue. Sympathetic nerve fibers bind to β2-AR on immune cells by releasing NE. VN downregulates proinflammatory factors by regulating macrophages, preserving the function of intestinal epithelial goblet cells and Paneth cells (Brinkman et al., 2019). When external stimulation occurs, the vagal afferent nerve and spinal afferent nerve act on the intestine and release neurotransmitters, such as CGRP, SP, VIP, etc., and then activate immune cells, such as mast cells, to regulate cytokine levels, injuring the gastrointestinal mucosa (Lai et al., 2017). The motor neurons and glial cells in ENS primarily constitute the submucosal plexus and intermuscular plexus. They regulate proinflammatory factors with intestinal resident immune cells to monitor the status of intestinal epithelial cells and promote intestinal homeostasis (Furness, 2012; Vergnolle and Cirillo, 2018). A variety of nerve and immune groups interact to maintain the integrity of the intestinal epithelial cell layer and the normal function of IECs.

ANS is composed of sympathetic nervous system (SNS) and PNS. It branches from CNS to form preganglionic neurons, and exchanges at nerve trunk or ganglions besides terminal effectors for SNS and PNS respectively, to regulate target organs. Postganglionic-sympathetic and parasympathetic fibers entering the intestinal wall form synapses with some enteric ganglion cells, transmit CNS information, and regulate gastrointestinal function. The SNS has a major inhibitory effect on gastrointestinal muscle and mucosal secretion, while the PNS has both excitatory and inhibitory effects on stomach, intestine and pancreas function, which is manifested as a more complex steady-state regulation (Verheijden and Boeckxstaens, 2018) (see Figure 1). Abnormal intestinal mucosal barrier function is closely related to the occurrence of intestinal diseases. Intestinal nerve regulation of intestinal immunity controls the function of the intestinal mucosal barrier. Early research found that patients with IBD exhibit ANS dysfunction, primarily manifesting as weakened VN function and enhanced sympathetic nerve function, accompanied by reduced functional neuron activity and reduced neurotransmitter release.

### Sympathetic Nervous System

Catecholamines released by the sympathetic nerve and the circulation of the adrenal medulla system affect intestinal lymphoid tissue, regulate intestinal immunity and affect intestinal mucosal barrier function (Cole et al., 2015). The axons of brainstem neurons project onto the presympathetic neurons of the spinal cord, releasing the primary neurotransmitter acetylcholine (Ach) at the ganglion site and releasing NE from the sympathetic neurons of the target organ. Mice lacking the Adra2ac receptor exhibit increased levels of NE throughout the body (Araujo et al., 2019). Upon activation of SNS at spleen, NE is released to bind α/β adrenergic receptor expressed on immune cells to facilitate neuroimmune communication. As a broad immunosuppressant, NE exerts an anti-inflammatory effect on innate immune cells, reducing the number of lymphocytes (Mracsko et al., 2014), increasing the number of immature monocytes, limiting the proliferation of specific antigen CD4^+^ T cells (Araujo et al., 2019), regulating the large number of cytokines produced by myeloid cells and lymphocytes (Mracsko et al., 2014; Agac et al., 2018; Willemze et al., 2019b) (see Table 1) and restricting the activation of T lymphocytes by inhibiting the interaction of APCs (see Table 1) (Agac et al., 2018). The catecholaminergic neurotoxin 6-OHDA treatment denervates the SNS and significantly reduces the migration of neutrophils and monocytes (Mousa et al., 2010).

**TABLE 1:** Summary of SNS-intestinal immunity manifestations in different diseases.

Disorder	Alterations of intestinal mucosal barrier	Intestinal immune/inflammatory responses	References
IBD	Depletion rate of goblet cells ↑	Inflammation grade of the colon↑; Inflammatory cytokines ↑	Willemze et al. (2018), Willemze et al. (2019a), Willemze et al. (2019b), Agac et al. (2018)
	AMP Reg3 γ expression↓		
Melanin precipitation	Intestinal mucosal immune activation	Intestinal mucosa HDC+ MC ↑	Yamate et al. (2011)
Stroke	Intestinal mucosal barrier permeability ↑	Inducing MMΦ to differentiate into inflammatory phenotypes	Liu et al. (2019)
	Intestinal bacteria translocation ↑	Activate CHAT+ T cell	
	TREM1 receptor ↑	Inducing CAIP pathway	

The SNS innervates all layers of the intestine and gut-associated lymphoid tissue, and the mesenteric lymph nodes (MLN) is innervated by sympathetic neurons projected by the superior mesenteric ganglion (Murray et al., 2019). In transcriptomics results of MMΦ, it was found that the Adrβ2 gene abundantly expressed and induces differentiation into an inflammatory phenotype (Gabanyi et al., 2016). Due to the anti-inflammatory effect of the SNS on innate immunity, the role of nerves and immunity in the treatment of IBD has been extensively studied (Willemze et al., 2018; Willemze et al., 2019a) (see Table 1). After administration of 6-OHDA or superior mesenteric sympathetic nerve transection in IBD mice, the concentration of catecholamines in the ileum was decreased, the score of colitis syndrome was significantly increased, levels of Th1 cytokines were increased, the “depletion rate” of goblet cells was significantly increased, and expression of the antimicrobial peptide Reg3 γ in the ileum was decreased (Willemze et al., 2019b) (see Table 1); *in vitro* experiments showed that β2AR deficiency further aggravated the inflammatory response of bone marrow-derived macrophages induced by LPS (see Table 1) (Agac et al., 2018). At the anatomical level, VN sparse distributing in the intestinal mucosa and submucosa, so activation SNS to initiate on the CAIP has been intensively studied. In this study, it was confirmed that the anti-inflammatory Choline acetyltransferase (ChAT)+ T cells in the intestinal mucosa and Peyer’s patches (PP) are increased by the activation of β2AR receptors, thereby improving the integrity of the intestinal mucosal barrier and symptoms of colitis (see Table 1) (Willemze et al., 2019a). Treatment by stimulating sympathetic nerve which specifically induces vagal afferent branch initiate anti-inflammatory pathway by prohibiting pro-inflammatory cytokines, activating anti-inflammatory cytokineswhich is the key mechanism of septic shock and colitis (Murray et al., 2019). Furthermore, stimulating SNS inducing histidine decarboxylase (HDC)+ mast cells proliferation thus inhibiting intestinal peristalsis (Yamate et al., 2011) (see Table 1). Liu et al. confirmed that expression of the TREM1 receptor (medullary triggering receptor1) on macrophages with a proinflammatory phenotype in the intestinal lamina propria was increased in a dose-dependent manner with NE, which increased the permeability of intestinal epithelial cells, increased the translocation of intestinal bacteria, and further aggravated brain injury (see Table 1) (Liu et al., 2019).

### Parasympathetic Nervous Systems

Parasympathetic innervation of the gastrointestinal tract depends on the VN (Verheijden and Boeckxstaens, 2018; Brinkman et al., 2019). The VN branches from the brainstem to the second segment of the transverse colon, supplying visceral organs with asymmetric motor neurons (Teratani et al., 2020) and abdominal branches into the stomach, pancreas, small intestine and colon. Approximately 80% of VN are afferent nerves, while 20% are efferent nerves. Intestinal VN efferent fibers are primarily distributed in the interganglionic nerve tracts and myenteric nerve plexus in the ENS, regulating fluid flow, hormone secretion, gastrointestinal peristalsis, and mucosal barrier function (Nijhuis et al., 2010). The VN, located in the dorsal motor nucleus, is involved in regulating gastric smooth muscle relaxation in Parkinson’s disease (PD) rats. Central dopaminergic neurodegeneration leads to a decreased density of intermuscular cholinergic neurons and a significant decrease in the concentration of Ach in extragastric muscles (Pellegrini et al., 2015).

VN does not innervate lymphatic organs, but participate in the regulation of intestinal barrier function through afferent and efferent fibers. The anti-inflammatory effects of VN efferent pathway are discussed in this paper. In animal models of sepsis, it was found that stimulating VN significantly inhibited inflammatory cytokine levels and improved animal survival rates, suggesting the CAIP (Kanashiro et al., 2017). VN’s terminal releases Ach, binds to the nicotinic acetylcholine receptor (nAChR) expressed on macrophages (Wang et al., 2003), downregulates the pro-inflammatory factor and inhibits the nuclear transmigration of the nuclear transcription factor NF-KB (Báez-Pagán et al., 2015). IBD is mainly manifested as a persistent inflammatory state, which can activate macrophage a7nAChR through the recruitment of Jak2 by nicotine, and activate the anti-inflammatory STAT3 and SOCS3 cascade signals to play their roles (de Jonge et al., 2005). Studies have found that VN cutting leads to communication barriers with mast cells, which cannot inhibit mast cell degranulation and protect intestinal mucosal barrier permeability, that is another mechanism of IBD (see Table 2) (de Haan et al., 2013). In addition, nicotine treated mature dendritic cell (DC) (Oyanguren et al., 2015) enhanced endocytosis and significantly reduced the secretion of pro-inflammatory factor (Nijhuis et al., 2010), and cholinergic nerve activation also had an effect on the “bias” of T cell differentiation (see Table 2) (Daoud et al., 2014; Bosmans et al., 2019; Teratani et al., 2020). β2-AR on DC stimulates ChAT+ T to enhance the epithelial AMP defense of the intestinal mucosa, which in turn prevents the composition and translocation of microorganisms (Dhawan et al., 2016). Interestingly, Bosmans et al. found that the improvement of Th2 inflammation by VN didn’t depend on the classic CAIP, but through the activation of cx3cr1^hi^ macrophages to produce immunosuppression, stimulate VN can inhibit MC to produce proteases and improve the membrane barrier transepithelial electrical resistance (see Table 2) (Bosmans et al., 2019). In addition to bone marrow derived macrophages, vagal nerve stimulation can also inhibit the activation of intestinal resident macrophages (Meroni et al., 2018), to prevent inflammation of the external muscle layer (Matteoli et al., 2014). Prucalopride, a 5-HT4R agonist, has also been shown to inhibit the activation of MMφ through the α7nAChR-mediated vagal pathway, preventing monocyte influx (Stakenborg et al., 2019).

**TABLE 2:** Summary of VN-intestinal immune manifestations in different diseases.

Disorder	Alterations of intestinal mucosal barrier	Intestinal immune/ Inflammatory responses	References
IBD	Goblet cell loss	Activated ChAT+ T cell	Willemze et al. (2019a)
	Crypt density ↓	Produce pro-inflammatory mediators	
	Intestinal mucosal barrier permeability ↑	Degranulation of MC	de Haan et al. (2013),
	Worsen TEER	Activate CX3CR1^hi^ macrophage	Bosmans et al. (2019)
		MC producing proteases Leukocyte ↑	
CD4^−/−^ mice	VN irritate AMP ↑	Activate Th1 immune response	Dhawan et al. (2016)
		M1 macrophage ↑	
Bacterial abdomina lSepticemia	Intestinal bacteria translocation ↑	VN regulate macrophage	Nullens et al. (2016)

The activation of VN afferent neurons leads to the activation of efferent motor neurons, which form synapses with sympathetic nerve cells in the abdominal ganglion, and innervate the spleen through postganglionic neurons. Activated spleen memory T cells produce cytokines to regulate anti-inflammatory immunity (Rosas-Ballina et al., 2011; Matteoli and Boeckxstaens, 2013). Nullens et al. found that administration of GTS-21, a selective a7nAChR agonist, regulated the VN-spleen macrophage circuit, improved intestinal mucosal barrier permeability, reduced bacterial translocation, and prevented further activation of gastrointestinal immune cells, providing a reasonable explanation for the treatment of multibacterial abdominal septicemia (Nullens et al., 2016) (see Table 2). When the intestinal mucosa encounters microbial molecules, Paneth cells and goblet cells secrete immunoglobulin and AMP, which assist in building the mucosal barrier function. Willemze et al. found that ChAT+ T cells detected in intestinal PP played a dual role in the colitis model. In the acute phase, leukocytosis activated the Th1 type immune response in the intestine and the mucosal barrier was destroyed; while in the remission phase of disease, after CD4+T cells specifically knocked out the CHAT gene, inflammation was poorly restored, M1 macrophage levels were increased, and goblet cells were depleted, suggesting that cholinergic T cells of non-neural origin are a special form of VN to rescue colitis in the later stages (Willemze et al., 2019a) (see Table 2).

## Intestinal Afferent Nerve-Immunity and Intestinal Mucosal Barrier Function

Nerves that perform impulse conduction from nerve endings to the center are afferent nerves, which are composed of sensory nerve fibers. Visceral sensory nerve fibers are often colocalized with autonomous sympathetic and parasympathetic nerves, but due to their special role in maintaining homeostasis, sensing danger and initiating protection, it is necessary to recognize their role in regulating intestinal immunity and function. Sensory neurons are equipped with specific types of ion channel sensors to complete functions, such as detecting changes in the environment. The activation of ion channels leads to the influx of Na+ and Ca2+, which converts the stimuli into electrical signals and completes the physiological process.

In a typical neural reflex circuit, the sensory neurons transmit peripheral changes to intermediate neurons of the CNS, while motor neurons on efferent nerves transmit signals to peripheral tissue. According to the literature, the gastrointestinal tract is primarily dominated by three kinds of sensory neurons: DRG (spinal nerve afferent), ganglion/cervical ganglion (vagus nerve afferent), and intrinsic primary afferent neurons (IPANs) (see Figure 1) (Lai et al., 2017). VN afferent nerve, the only cranial nerve innervating thoracic and abdominal sensory responses, is an important part of the neuro-immuno-endocrine axis. The afferent cell body of the spinal cord enters the spinal cord along the DRG, forms synapses in the dorsal horn, or continues upward, forming synapses in the brainstem to transmit information to the center (Spencer et al., 2016).

### Spinal Afferent Nerve

After injecting anterograde markers into live mice, spinal cord afferents in the large intestine were found to be primarily distributed in the muscular-enteric ganglion, submucosa, and inner ring muscle layer (Spencer et al., 2016; Yu et al., 2016). Due to the large distribution of spinal cord afferents from the lumbosacral region in the colon, it has become a major node in the study of pain perception and inflammation in the colon.

The ability to perceive painful stimuli is mediated by nociceptors, and their role is to minimize damage as much as possible. The nerve endings that receive signals secrete many neurotransmitters, such as calcitonin-related gene peptide (CGRP), substance P (SP), tachykinin, nitrogen oxide (NO), and cholecystokinin, which combine with the corresponding receptor to exert immune regulatory effects. Nociceptive neurons have an effect on systemic immunity, for example, by expressing the relevant receptors and binding to cytokines (de Macedo et al., 2019) and chemokines (Chen et al., 2020) produced by leukocytes to indirectly detect the host state. TRPV1 is a representative nociceptive neuron, secreting the anti-inflammatory CGRP which specifically downregulates the TNF-ɑ level after activation, exerting a synergistic anti-inflammatory effect with macrophagesand DCs, maintaining intestinal mucosa homeostasis (Assas et al., 2014). After nociceptors activation, macrophages are launched, and pathological pain is enhanced (Chen et al., 2020). Similarly, nociceptors also play an important role in the conversion of cytokines involved in Th1/Th17- and Th2-type immunity (Kashem et al., 2015; Pinho-Ribeiro et al., 2018; Crosson et al., 2019).

Nociceptors sense pathogenic bacteria in the intestine and exert immune regulation in advance. Lai et al. found in an ileal inflammatory disease model simulated by Salmonella infection, that TRPV1 and Nav1.8-type nerves directly regulate levels of APCs in the intestinal epithelial cell layer to limit damages in the intestinal mucosal barrier and disturbances of intestinal flora caused by the pathogenic bacteria invading (see Table 3) (Lai et al., 2019). TRPM8, a cold sensation receptor or can be activated by menthol and icilin,, has been found to directly inhibit the proliferation and activation of myeloid immune CD11c+ DCs after targeted ablation (see Table 3) (de Jong et al., 2015). Although TRPM8^−/−^ mice are more likely to experience colitis, studies have found that loss of TRPM8 can not cause intestinal barrier dysfunction or epithelial cell tissue destruction (Ramachandran et al., 2013). In bacterial colitis diseases, ILCs activate IL-22 and enhance the bactericidal ability of IECs to produce AMP. Recent studies have found that TRPV1 plays a cooperative role in it; TRPV1^−/−^ mice IL-22 and T cell recruitment are reduced in the colon, and the gut flora is transferred to surrounding organs (Ramirez et al., 2020) (see Table 3).

**TABLE 3:** Summary of Afferent nerve-intestinal immunity manifestations in different diseases.

Disorder	Alterations of intestinal mucosal barrier	Intestinal immune/inflammatory responses	References
IBD	Ileum M cell ↑	Inhibit CD11c+ DCs proliferation;	Lai et al. (2019), de Jong et al. (2015)
	Inhibit bacteria translocation	IL-22 level ↑	Ramirez et al. (2020)
	Secretion AMP ↑	T Cell recruitment ↑	
	Activate HPA axis release GC		Bonaz et al. (2017)
IBS	Injury to gastric mucosal tissue	Degranulation of mast cell	Xu et al. (2018)

Changes in the nature of sensory neurons produce peripheral pain (Salameh et al., 2019), and IBD-related sequelae include functional symptoms of remission and increased anxiety (Akbar et al., 2008). The pain associated with gastrointestinal inflammatory diseases is often accompanied by mast cell infiltration. Abnormal neuroimmunomodulation in IBS leads to visceral hypersensitivity. Mast cells release tryptases bined with PAR2 receptors to release the neurotransmitters CGRP and SP, which in turn aggravate mast cell activation and worsen colonic inflammation. In acute gastric mucositis, TRPA1 is a temperature-sensitive receptor that activates and releases SP in large quantities, leading to submucosal mast cell degranulation, mediating neurogenic inflammation and damaging gastric mucosal tissue. HC-030031, a specific antagonist of TRPA1, reverses this nociceptive stimulation and plays a protective role (Xu et al., 2018) (see Table 3).

### Vagus Afferent Nerve

Afferent fibers of the vagus nerve originate in different intestinal layers, but they all terminate in the solitary nucleus (NTS). The VN afferent branch that connect the dorsal motor nucleus (DMV) with the NTS is the site where visceral sensory information is collected and integrated (see Table 3) (Bonaz et al., 2017). Persistent chronic inflammation of IBD induces TNF-α, IL-6, etc. to bind correspondant receptors on the vagus neuron, to activate the Hypothalamic-Pituitary-Adrenalin axis to release glucocorticoids to suppress inflammation. In addition, the vagal afferent nerve transmits information to the efferent nerve to exert anti-inflammatory effects of CAIP and is an important method to treat IBD.

Oral tolerance is an important way for intestinal mucosal immunity that maintain homeostasis. The latest research has found the liver VN sensory afferent branch senses the intestinal microenvironment, transfers sensory input to the NTS, and terminates in the vagal parasympathetic nerve and intestinal neurons, forming a liver-brain-gut network which is a novel Neuroimmune modulatory mechanism (Teratani et al., 2020). Interruption of the left vagus afferent nerve of the liver reduces the number of pTregs (peripheral regulatory T cells) that activate APCs, resulting in the increased susceptibility to colitis.

## Intestinal Intrinsic Nerve-Immunity and Intestinal Mucosal Barrier Function

Intestinal innervation is provided by the ENS, an interconnected network of neurons and glial cells that controls intestinal movement, fluid exchange on the mucosal surface, blood flow, and intestinal hormone secretion (see Figure 1) (Furness, 2012; Keita and Söderholm, 2010). Under the physiological and pathological backgrounds, IPANs and intrinsic effector neurons control mucosal function, blood flow, and immune cell migration (Vergnolle and Cirillo, 2018) (see Figure 1). Intestinal inflammation caused by the autoimmune of EGC is a possible pathological mechanism of Crohn’s disease, suggesting the communication between ENS and intestinal immunity (Cornet et al., 2001).

### Enteric Neuron-Immunity and Intestinal Mucosal Barrier Function

Different locations, branches, and identities of neighboring cells create the richness and specificities of neurons. Single-cell sequencing further divides enteric neurons into more natural nitrate energy (i.e., expresses neuronal nitric oxide synthase Nos1, ENT1-3) and cholinergic functions (i.e., expresses chat and Slc5a7, ENT4-9) (Zeisel et al., 2018). ENS neurons secrete large amounts of neurotransmitters and neuropeptides, such as Ach, NE, NO, Vasoactive Intestinal Peptide (VIP), and SP. The ENS is an important component of the intestinal innate immune response. Intestinal-specific IL-18 neurons can drive the production of AMP in goblet cells and protect against *Salmonella typhimurium*, which is very important for coordinating homeostasis of the mucosal barrier (Jarret et al., 2020). The early stage in diabetic rats (BB-DP) is characterized by high permeability of the intestinal mucosal barrier. As time goes on, mucosal immunity is activated, and myenteric nitrogenous neurons are lost and exhibit loss of function, which eventually leads to intestinal dyskinesia (see Table 4) (Vanuytsel et al., 2014). Gastrointestinal dysfunction, one of the early symptoms of PD, has been observed in animal models in which CD4^+^ T cells drive inflammatory responses in the intestinal mucosa, leading to a decrease in the number of dopaminergic neurons in the myenteric and submucosal plexuses (see Table 4) (Campos-Acuna et al., 2019). In addition, amyloid β deposition and overexpression of phosphorylated Tau protein in intestinal myenteric neurons of neurodegenerative disorder Alzheimer’s disease (AD) mice, significantly increased the number of CD68^+^ macrophages in the ileum and further led to the loss of intramuscular nitrogenous and cholinergic neurons in the ENS (see Table 4) (Han et al., 2017). Intestinal cholinergic neurons also provide treatment ideas for intestinal inflammation and damage to the mucosal barrier by activating immunity. Stanley et al. found that in gastrointestinal disorders associated with stroke, due to a loss of ileal cholinergic ChAT+ neurons, which stimulate the development of proinflammatory immunity, increased barrier permeability led to intestinal bacterial translocation and secondary infection (Stanley et al., 2016) (see Table 4).

**TABLE 4:** Summary of ENS-intestinal immunity manifestations in different diseases.

Disorder	Alterations of intestinal mucosal barrier	Intestinal immune/inflammatory responses	References
BB-diabetes prone	Intestinal mucosal barrier permeability ↑	Myeloperoxidase abundantly expressed in neutrophils	Vanuytsel et al. (2014)
	Nitrogen neurons ↓		
PD	Dopamine neurons ↓	Activate CD4+ immune response	Campos-Acuna et al. (2019)
AD	Nitrogen and cholinergic neuron ↓	CD68+ macrophage ↑	Han et al. (2017)
Stroke	Bacteria translocation and infection	IL-17 + y&T ↑	Stanley et al. (2016)
IBD	VIP neuron regulating AMP;	ILC3 produce IL-22 ↑	Chandrasekharan et al. (2013), Ballout and Diener (2019), Talbot et al. (2020),
	Claudin-2 ↑	Activate MC; secretion of negative ions of cytokines was impaired; inhibit the growth of IL-1B and IL-10;	Bessac et al. (2018), Kermarrec et al. (2018)
	Intestinal neuron loss	Secrete IL-17 promote intestinal homeostasis	
	Inflammatory cytokines and histamines pass through freely	Activate MC	
	Antiapoptotic activity of colonic cells ↓	RET induce ILC3 to secrete IL-22	Ibiza et al. (2016)
	GDNF induce intestinal TJ ↑		

Intestinal resident macrophages are specific subtypes of macrophages that form populations in the intestine with peripheral monocytes, myeloid cells and self-sustaining macrophages (Na et al., 2019). An imbalance in macrophage subsets is closely related to abnormal submucosal vascular networks and neuronal degeneration in the ENS. After oral administration of SPIB mice were infected, the signal of the β2ARs in MM φ caused activation of sympathetic SMG ganglia and release of NE (Gabanyi et al., 2016); vagal afferent nerves can regulate the anti-inflammatory effect of MMφ (Matteoli et al., 2014). MMφ can quickly respond to pathogens that infect the intestine and limit neuron loss caused by infection through the MMφ-β2 adrenaline arginase 1-polyamine axis (Matheis et al., 2020). Disruption of the M1/M2 macrophages is accompanied by the loss of enteric neurons and enteric neural stem cells and interferes with epithelial barrier integrity (Becker et al., 2018). The development of MMφ is controlled by the Colony Stimulating Factor (CSF1) expressed by intestinal neurons. The number of NO neurons in the intestine of CSF^−/−^ mice was increased, and the lack of bone morpho protein 2 expression in MMφ led to the immaturity of myenteroneurons (Cipriani et al., 2019). Macrophage depletion after anti-CSF1R treatment affected the differentiation of Paneth cells and impaired the differentiation of intestinal epithelial cells.

#### Intestinal Motor Neuron

Motoneurons secreted in the intestine, such as VIP+ neurons and Neuropeptide Y (NPY)+ neurons, regulate a variety of immune cells. VIP+ neurons mediate VIP secretion, monitor intestinal epithelial status by regulating lymphocytes (Talbot et al., 2020) (see Table 4), and regulate mononuclear phagocytic cells (MNPs) (Buckinx et al., 2017) and their secreted cytokines, tilting immunity towards the Th2 type. VIP-sensitized DCs induce Treg production and restore immune tolerance. Talbot et al. found that IL-22 produced by ILC3 can be inhibited by VIP neurons that express VIPR2 receptors, regulate AMP levels, and promote normal function of the intestinal barrier; VIP neuron activation also promotes ileal epithelium colonized bacteria grow, enhances lipid absorption, and forms a dynamic neuroimmune circuit (Talbot et al., 2020) (see Table 4). In mice with colitis, NPY neurons are upregulated, resulting in increased intestinal permeability and induction of claudin-2. Administration of TNF inhibitors reverses NPY expression, reduces inflammation and oxidative stress, and prevents increased epithelial permeability (see Table 4) (Chandrasekharan et al., 2013).

Mast cells cocultured with intestinal secretory motoneurons significantly increase neuronal activity, and the latter also induce degranulation of SP and other neuropeptides in mast cells (see Table 4) (Ballout and Diener, 2019). Destruction of the intestinal mucosal epithelium in IBD rats leads to the free passage of proinflammatory cytokines, histamine and other secretory mediators is the primary factor leading to chronic intestinal inflammation, in which the impairment of cytokine anion secretion is caused by the numbers of motor neurons decreased which activated via mast cells, thus affects intestinal epithelial barrier function (Ballout and Diener, 2019) (see Table 4).

#### Intestinal Endogenous Sensory Neuron

There are also sensory neurons on the wall of the gastrointestinal tract that do not require complex pathways, such as central or peripheral nerves, to dominate nerve reflexes and motor patterns. With the discovery of Dogiel II neurons as sensory neurons in the myenteric plexus in the small intestine of mice, IPAN have been found to regulate intestinal immune-motor function (Mao et al., 2006). In colitis driven by Th2 type T cells, smooth muscles are contracted excessively, while colitis Th1 causes muscles to insufficiently contract (Shea-Donohue et al., 2012; Bessac et al., 2018) (see Table 4). Except for a few mesenteric spinal cord neurons, intestinal sensory neurons do not project to the CNS and are less likely to participate in the sensation produced by the intestinal wall. Moreover, microbes can be used as intermediates to connect neuroimmunity. Bacteria in the intestinal cavity can excite IPAN, but when the intestinal epithelial barrier is destroyed, the microflora can also be presented to the intestinal submucosal immune cell population by specific cells, stimulating the development of gut-associated lymphatic tissue, maintaining the tolerance and dynamic balance of immune cells, suggesting that the intestinal mucosal barrier treatment of IBD against microorganisms is also very important (Jamar et al., 2020).

The broken of intestinal mucosal barrier or the relieve of related immune responses in gastrointestinal diseases may cause mucosal inflammation and increased afferent sensory signals, leading to abdominal pain (Wells et al., 2017). Chang et al. believe that administration of drugs can restore expression of TRPV1 in the duodenum and decrease the proinflammatory cytokines level, which may regulate mucosal barrier permeability in the treatment of FD (Chang et al., 2017).

### Enteric Glial Cell-Immunity and Intestinal Mucosal Barrier Function

EGC networks are the supporting structures of the ENS, which are distributed at various levels in the intestine, transmitting neurotransmitters and processing information (Aube et al., 2006). Expression of the glial markers GFAP and Sox-10 is increased in colon tissue of PD patients. A large number of studies have shown that EGC produces nutritional factors (e.g., GDNF) that have strong anti-apoptotic activity, protect the IEC barrier and support intestinal barrier permeability (Yu and Li, 2014; Meir et al., 2015; Bauman et al., 2017). In IBD mice, excessive proliferation of EGCs leads to abnormal inflammatory activation of related immune cells, which also mediates intestinal damage (De Filippis et al., 2011). Among them, EGCs communicate with mast cells in two ways, and mast cell activation can promote the activation of intestinal glial cells and macrophages, resulting in intestinal mucosal injury and neuronal reduction (Nogueira et al., 2017). However, recent studies have found that GDNF derived from EGCs also significantly inhibits mast cell degranulation and attenuates the inflammatory state (Xie et al., 2020). When EGCs are exposed to immune cytokines such as IL-1β or low levels of IL-10, they can prevent proliferation (Bessac et al., 2018) and secrete IL-17, promoting T cell survival (see Table 4) (Kermarrec et al., 2018). Intestinal ILC3 subsets express neuromodulatory receptor tyrosine kinase (RET), which is activated by GFL, a ligand of the GDNF family derived from EGCs. Activated RET induces ILC3s to secrete IL-22 and causes expression of tight junction proteins in epithelial cells, which effectively promotes intestinal homeostasis (Ibiza et al., 2016) (see Table 4).

## Intestinal Microflora Regulate Neuro-Immunity and Intestinal Mucosal Barrier Function

The intestinal flora include thousands of species of eubacteria, archaea, eukaryotic microorganisms and noncellular structural viruses. As the field of gut microbiota continues to gain knowledge, we re-examine its role in nervous system diseases. Enterobacteria and their metabolites can directly maintain the integrity of the intestinal epithelial barrier by regulating the growth and differentiation of IECs and the expression of tight junction proteins (Pellegrini et al., 2018). Recent studies have shown that intestinal microorganisms regulate the composition and function of the mucous layer, which is a component of the innate immune intestinal mucosal barrier and participates in reducing the activation of the immune system under intestinal epithelial cells by antigen and bacterial exposure. Both symbionts and pathogens can degrade and use mucin as an energy source and attachment site to increase colonization, but pathogens can also cause infection through intestinal leakage (Paone and Cani, 2020). Furthermore, Microbial-related molecular patterns expressed in intestinal microorganisms can activate Toll-like receptors on innate immune cells and secrete anti-inflammatory mediators to maintain intestinal immune tolerance. And intestinal dysbacteriosis affects differentiation of CD4+ and CD8+ T cells and induces an adaptive immune response (Benakis et al., 2016; Muñoz et al., 2019). With the colonization of microorganisms in the gastrointestinal tract, the mucosal immune system gradually matures and induces 5-HT to promote the development and function of ENS (primarily in EGCs) (De Vadder et al., 2018). Studies have shown that intestinal microbes regulate exogenous sympathetic neurons in the intestine (Muller et al., 2020). Bacterial pathogens also directly activate DRG sensory nerves in the colon, leading to intestinal infection and aggravating the pain of large intestine expansion (Ramirez et al., 2020). Microorganisms and their metabolites can activate the vagus system by activating enterochromaffin cells to release a variety of neuropeptides and neurotransmitters, regulating the gut-brain axis (De Vadder et al., 2018; Pellegrini et al., 2018).

However, gradully attention has been given to the regulation of intestinal microflora, neuroimmune regulation and the dynamic balance between intestinal mucosal epithelium. In IBD, imbalances in the intestinal microbiota lead to barrier leakage that activates the immune response. The sampling mode of special epithelial M cells in the distal ileum is destroyed, and intestinal microorganisms can enter and exit freely to enhance the immune response (Lai et al., 2019). The increased permeability of the intestinal mucosal barrier regulated by the sympathetic nerve leads to intestinal bacterial translocation, which is an important mechanism of pathogenesis of secondary pulmonary infection after stroke. After MCAO for 24 h, the abundance of microorganisms in mice was significantly decreased in the colon and ileum, and enterobacteria, such as Lactobacillus, actinomycetes, Lactococcus and Clostridium, were enriched in the lung (Stanley et al., 2016).

## Conclusions and Perspectives

The gut barrier is a special district that responds and interacts with different intestinal stimuli and microbiomes. Intestinal inflammatory diseases, typical clinical features of remission and progressive gastrointestinal dysfunction, cause nonspecific inflammation and intestinal barrier tissue damage (Miner-Williams and Moughan, 2016). Pathogenesis involves complex interaction disorders among multiple factors, and innate and adaptive immune responses have become key factors. Gut-brain dual signaling plays an important role in the maintenance of intestinal barrier function. Recently, the use of targeted cell specificity and new sequencing, imaging and analysis tools in the study of mucosal barrier function in gastrointestinal diseases has revealed unknown neuroimmune regulatory mechanisms.

At present, we focus on the intermediate links of common clinical diseases such as stroke, neurodegenerative diseases (Parkinson, dementia), diabetes or constipation—intestinal function destruction, intestinal flora shift, and the excessive activation of intestinal immune response play a crutical role in it. Especially, the intestinal bacterial translocation has been proved to be an important disease mechanism in the secondary pulmonary infection after CNS injury. The permeability of the intestinal mucosal barrier is increased, the balance of microorganisms in the intestinal lumen is broken and the migration occurred. In this paper, we summarized the research progress of intestinal afferent nerves, efferent nerves, and ENS in regulating intestinal mucosal barrier function through immune communication. The function and quantity of innate immune cells and T lymphocytes are regulated by SNS release of NE, which can regulate the function and quantity of innate immune cells and T lymphocytes, regulating the function of intestinal mucosal epithelial cells to produce AMP and intestinal mucosal barrier permeability. VN primarily manipulates the phenotype of macrophages through CAIP downregulation of the production of the proinflammatory factor TNF-α, rescuing the “depletion rate” of goblet cells, and maintaining the normal permeability of the intestinal mucosal barrier. Exogenous afferent nerves, including the VN and spinal cord afferent nerve, colocalize with other efferent nerve terminals in the intestine to release a series of neurotransmitters, neuropeptides and cytokines through nociceptive stimuli, which can interfere with intestinal macrophages, DCs and neutrophils to maintain homeostasis but can also activate mast cell degranulation and then damage the integrity of the mucosal barrier. However, the beneficial effects of nociceptors on the intestinal mucosal barrier are lacking. In addition, gut-brain axis signals may be affected by delayed mucosal immune activation, resulting in an increase in afferent sensory signals and abdominal symptoms. Patients with gastrointestinal diseases often show increased susceptibility of the viscera to multiple stimuli. On the one hand, visceral afferent signals are abnormally amplified at the level of the spinal cord and brain; on the other hand, the sensitivity of receptors in the intestinal wall to multiple stimuli is abnormally increased, and the threshold is decreased. Secretory motor neurons and enteric glial cells in the intestinal intrinsic nervous system primarily communicate with innate lymphocytes to inhibit the production of the proinflammatory factor IL-22 and restrain the degranulation of mast cells, which in turn acts on tight junction protein expression between intestinal epithelial cells and restores intestinal epithelial barrier function. In conclusion, we regard the neuroimmune interaction of the intestine and the complementation of the nervous system as affecting the function of the gastrointestinal mucosal barrier.

Gastrointestinal dysfunction and intestinal mucosal barrier damage are early symptoms, representing physiological and pathological links of lung infection secondary to CNS injury and neurodegenerative diseases. The mechanism of the signaling pathway from the gut microbiota to the brain were discussed in detail in this paper. Although the many studies on the intestinal nerve and intestinal adaptive immune response upon microorganisms and their metabolites changing, the permeability of the gut mucosal barrier regulated by neuroimmune mechanism are still few. It will be possible to supplement the key role and causal relationship of neuroimmune in the microbial-gut-brain axis to provide a new direction for disease prevention and treatment.
